# Cystic fibrosis–related metabolic defects: crosstalk between ion channels and organs

**DOI:** 10.1172/JCI182329

**Published:** 2024-07-01

**Authors:** Sunder Sims-Lucas, Eric S. Goetzman, Thomas R. Kleyman

**Affiliations:** 1 Department of Pediatrics, and; 2 Department of Medicine, University of Pittsburgh School of Medicine, Pittsburgh, Pennsylvania, USA.

## Abstract

Cystic fibrosis is a debilitating disease characterized by a poor medical prognosis due to devastating lung injury. Recent medical advances targeting the major genetic mutation ΔF508 of the cystic fibrosis transmembrane conductance regulator (CFTR) protein have dramatically increased the lifespan of patients with this mutation. This development has led to major changes in the field and has pushed research beyond the ion transport nature of cystic fibrosis and toward multiorgan physiological reprogramming. In this issue of the *JCI*, Bae, Kim, and colleagues utilized a large animal pig model prior to the onset of disease. They revealed metabolic reprogramming and organ crosstalk that occurred prior to disease progression. These findings provide paradigm-shifting insight into this complex disease.

## Trikafta propels a new era in CFTR research

**Figure 1:**
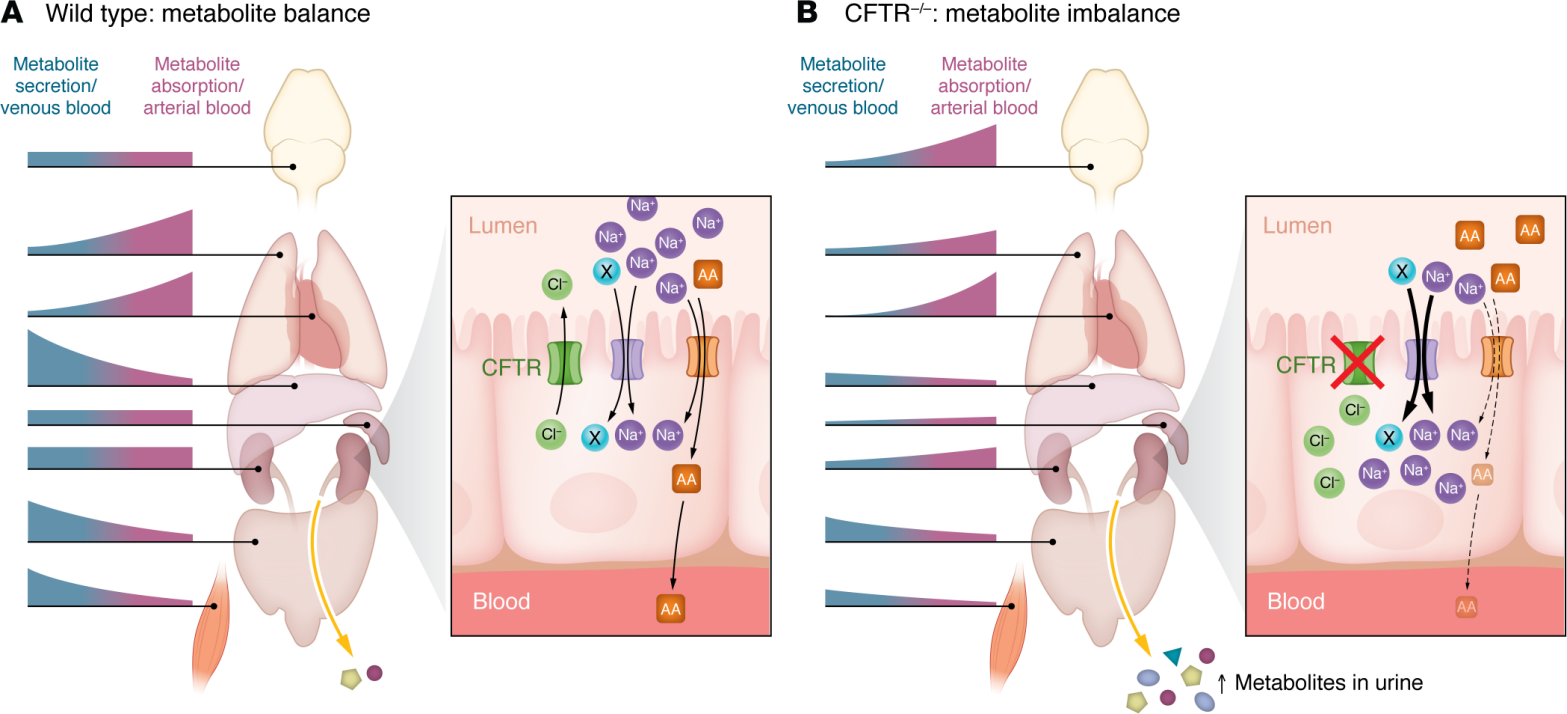
CFTR contributes to whole-body metabolite balance. (**A**) Bae et al. (12) show that in wild-type pigs, intestine, liver, and muscle primarily secrete metabolites, while other organs such as the lung and heart primarily take up metabolites. The healthy kidney plays a key role in metabolite homeostasis, taking up amino acids (AA) and other metabolites when concentrations are high and releasing them when concentrations are low. Metabolite loss to the urine is minimized by the absorption of filtered metabolites in proximal tubular segments of the nephron. This process is maintained by numerous Na^+^-dependent (or H^+^- dependent) transporters on the apical surface of proximal tubular cells. (**B**) In CFTR^–/–^ pigs, the inter-organ balance of metabolites is disturbed. The CF kidney presumably has disrupted ion balance across the tubular epithelium due to loss of the CFTR chloride transporter. Hypothetically, activation of a yet-to-be identified Na solute (solute X) cotransporter, or downregulation of the Na K-ATPase, leads to an increase in the intracellular Na^+^ concentration in proximal tubular segments of CF kidneys and disrupts the transport of amino acids and other metabolites. As a result, CF kidneys would have disordered metabolite homeostasis, which increases metabolite loss to the urine.

Cystic fibrosis (CF) is a genetic condition that affects the cystic fibrosis transmembrane conductance regulator (CFTR) protein (1, 2). Patients with CF have defects in the movement of chloride, sodium, and water in and out of cells (3). In the lung epithelium, this defect dramatically increases the viscosity of the mucus, resulting in life-threatening blockages (4). While there are over 2,000 mutations that are known to cause CF, the most common mutation is the ΔF508 mutation that is seen in approximately 90% of patients (5). In the United States, approximately 1 in 30 individuals are CF carriers, and it is estimated that there are currently over 160,000 people living with CF (6). Several decades ago, the life expectancy for patients with CF was just 30 years of age, and treatments were limited (7). However, recent medical breakthroughs, particularly the FDA approval of Trikafta in 2019 (VX18-445-104, ClinicalTrials.gov ID: NCT04058353), have increased life expectancy for patients with CF into the fifties and is continuing to rise, with some patients now living into their eighties (7). Trikafta is the first approved CF medication that affectively treats patients with the ΔF508 variant and has been approved for use in patients as young as two years of age (8). This combination of three drugs (elexacaftor, ivacaftor, and tezacaftor) targets the defective CFTR protein, modulating its ion channel activity and half-life at the cell surface, and is particularly effective at improving the function of the ΔF508 variant. The dramatic improvements in health span spurred by Trikafta have ushered in a new era of CF research, and a shift has been made to studying the secondary non-lung phenotypes that were once a minor concern due to the high mortality caused by the lung phenotype. Other organ systems that are now areas of research focus include the liver, pancreas, sinuses, intestines, and kidneys, among others (9). The kidneys are of consequence, as they are heavily enriched in ion channels (10). Defective ion exchange due to CF dramatically changes the metabolic output of the kidney (Figure 1) and the crosstalk of this with other organ systems (11). Thus, the focus of the field has shifted from the traditional ion transport and trafficking of CFTR and toward the study of integrated physiology. In this issue of the *JCI*, Bae, Kim, and authors (12) explored the metabolic profile of organs that were subjected to cystic fibrosis mutations by studying a large animal model of cystic fibrosis prior to the onset of disease. The findings suggest that metabolism and crosstalk among organs have roles in driving dysfunction in CF.

## Inadequate animal models have limited physiological research of CFTR

Studies in CF and CFTR mutations have been challenging, and models that accurately reflect the human condition have been inadequate. There are many facets that have hampered the experimental models including the various comorbidities in humans that confound many of the findings, including diabetes, gastroesophageal reflux, and inflammatory bowel disease, among others (6). Further, in CFTR-deficient rodents, many of the pathological abnormalities observed in patients with CF are absent, and thus the utility of these models has been very limited (13). The study by Bae, Kim, and colleagues (12) utilized an exciting large animal porcine model, which is known to recapitulate much of the pathology seen in patients with CF (14). However, the authors took an exciting and innovative angle by interrogating the various metabolites that were altered in young animals prior to the onset of observable disease. They also utilized a unique system, whereby they performed blood draws from arteries and veins from the specific organs to determine which metabolites were generated and utilized in individual organs and, comparatively, which metabolites were affected in relation to organ crosstalk. In this way, the authors concluded that the CFTR mutation drove developmental phenotypes that were specific to metabolism and that had occurred prior to the onset of observable disease. These findings are groundbreaking for the field, as they open up and diversify the study of CF and CFTR to incorporate physiological changes observed in the metabolites in CF piglets (12).

## Arteriovenous metabolomics reveal CFTR effects on metabolite homeostasis

The inter-organ exchange of glucose is paramount to survival. During periods of fasting or metabolic stress, the liver and kidneys convert carbon from amino acids, lactate, and glycerol into glucose and release it for use by the brain and periphery. Patients with CF display perturbations in glucose homeostasis and are at high risk for diabetes (15). Previous work on the pig model showed that even newborn CF piglets have defects in glucose handling (16). Those studies were all done by following systemic glucose concentrations. Bae et al. (12) used arteriovenous metabolomics to shed further light onto this phenomenon. First, they saw that while wild-type pig liver and kidneys both had a net release of glucose, CF liver and kidneys did not. The release of glucose by CF organs was near zero, and in some animals, glucose was being taken up rather than released. Then, the authors looked at the correlation between circulating glucose levels and the rate of uptake/release by liver and kidneys. These two organs are responsible for sensing circulating glucose levels and maintaining them within a narrow window by either taking up excess glucose or secreting glucose when levels drop. In wild-type piglets, Bae, Kim, and colleagues observed a remarkably tight inverse correlation between circulating glucose concentrations and the rate of kidney glucose release. As circulating levels dropped, the kidneys secreted increasing amounts of glucose. Strikingly, in CF piglets, there was no correlation at all between circulating glucose concentrations and kidney glucose release, suggesting defects in glucose sensing, transport, or both. It is of interest to note that glucose absorption in the kidney occurs primarily in the first two segments (S1, S2) of the proximal tubule, and that this uptake is dependent on cotransport with Na^+^ through Na^+^-glucose cotransporters (SGLTs). Recent advances in segment-specific mapping of the nephron transcriptome indicate that CFTR is also expressed in the S1 and S2 segments of the proximal tubule, placing it in physical proximity to SGLT2 (17, 18). Through mechanisms that are not well understood, CFTR is known to blunt the activity of epithelial Na^+^ channels, such that deletion of CFTR leads to enhanced Na^+^ transport in specific experimental systems (19, 20), although the role of ENaC in CF pathogenesis is still unclear (21). It is tempting to speculate that loss of CFTR in S1 and S2 proximal tubular segments leads to SGLT dysregulation.

Glucose homeostasis can be complicated due to the actions of powerful hormones such as insulin and glucagon. Much simpler homeostatic mechanisms apply to amino acids. Like glucose, amino acid levels in the blood are maintained within a narrow range. But unlike glucose, amino acid homeostasis requires no hormones and is achieved simply through the law of mass action. In other words, when amino acid concentrations are higher inside cells than outside, amino acids are transported out, and vice versa. In the kidney of the newborn CF pig, it turns out that even this much simpler form of metabolite homeostasis is disturbed. Whereas wild-type piglet kidneys maintained a negative correlation between blood amino acids and uptake/release rates, i.e., taking up amino acids when blood levels were high and releasing them when blood levels were low, CF piglet kidneys tended to do the opposite. As a result, CF piglets lost more amino acids into the urine. Although amino acid homeostasis is driven by mass action, amino acids cannot cross membranes on their own and require facilitated transport. The absorption of filtered amino acids occurs in the S1, S2, and S3 segments of the proximal tubule by a family of luminal membrane amino acid transporters (22, 23), where amino acids are largely cotransported with Na^+^ or H^+^ (24). A theme emerges in which loss of CFTR at the apical surface of cells in the S1 and S2 segments of the proximal tubule appears to dampen the activity of Na^+^- or H^+^-coupled amino acid cotransporters. While the mechanisms by which this occurs are unclear, Bae et al. (12) did not report evidence of a decreased transporter message or protein expression and suggest that changes in the electrochemical gradients for Na^+^ or H^+^ were responsible for the reduced absorption of filtered amino acids. Additional studies are needed to determine whether changes in the Na^+^ or H^+^ concentrations in the proximal tubular lumen or within proximal tubular cells occur in the absence of CFTR. For example, a higher intracellular Na^+^ concentration could dampen apical uptake of metabolites and potentially affect basolateral transport back to the circulation. The result would be the lack of relationship between circulating metabolite levels and the rate of uptake/release, as was observed in the work by Bae et al. for so many metabolites in CF piglet kidneys (12). The balance of metabolites across compartments in CF kidneys would be less responsive to metabolite concentrations and instead becomes tied to the intracellular concentrations of Na^+^ and other cations (Figure 1).

## Conclusion and future perspectives

For patients with CF, improved therapies mean an improved health span in terms of length and quality. Previously, the life-threatening severity of lung symptoms demanded the bulk of the attention of clinicians and CFTR researchers. Now, in a new era of CF science, we have the luxury of diverting attention to solving longstanding mysteries of CFTR biology such as derangements to metabolism. The work of Bae, Kim, and authors (12) represents a critical first step toward unraveling the mechanisms by which ion homeostasis affects metabolism across the whole organism.

## Version 1 07/01/2024

Electronic publication
